# Supplementary material for: Taxonomic and Phylogenetic Studies of Two Brackish Pleuronema Species (Protista, Ciliophora, Scuticociliatia) from Subtropical Coastal Waters of China, with Report of a New Species
Source: Microorganisms. 2023 May 27;11(6):1422. doi: 10.3390/microorganisms11061422 (PMC10304819; doi:10.3390/microorganisms11061422)
Supplement: Supplementary file 1 [file microorganisms-11-01422-s001.zip › microorganisms-2394265-supplementary.pdf]

Table S1. GenBank accession numbers of all sequences included in phylogenetic analyses.

Species	Accession no.	Species	Accession no.
**PLEURONEMATIDAE**		*Pleuronema* cf. *setigerum*	FJ848875
***Pleuronema ningboensis* n. sp.**	**OQ591738**	*Pleuronema marinum*	KF206428
Pleuronema orientale	**OQ591739**	*Pleuronema* sp.	FJ848876
Pleuronema puytoraci	KF840520	*Pleuronema sinica*	EF486864
Pleuronema orientale	KF206429	*Pleuronema parawiackowskii*	KT033423
Pleuronema paraorientale	OL654419	*Pleuronema wiackowskii*	JX310016
Pleuronema foissneri	OL654416	*Pleuronema czapikae*	EF486863
Pleuronema parasalmastra	OL654418	*Pleuronema* sp.	JX310017
Pleuronema parasmalli	OL654417	*Schizocalyptra sinica*	FJ156106
Pleuronema setigerum	FJ848874	*Schizocalyptra aeschtae*	DQ777744
Pleuronema coronatum	AY103188	*Schizocalyptra* sp.	FJ848873
Pleuronema coronatum	HM140396	*Schizocalyptra similis*	EU744177
Pleuronema coronatum	JX310018		
Pleuronema coronatum	JX310014	**OUTGROUP**	
Pleuronema elegans	KF840518	*Falcicyclidium plouneouri*	FJ868181
Pleuronema binucleatum	KT033424	*Falcicyclidium fangi*	FJ868185
Pleuronema grolierei	KF840519	*Acucyclidium atractodes*	FJ868182
Pleuronema setigerum	JX310015	*Hippocomos salinus*	JX310012
Pleuronema paucisaetosum	KF206430	*Wilbertia typica*	FJ490551

The two newly submitted sequences in the present work are in bold.
